# Correlation between the number of false positive variants and the quality of results using Ion Torrent PGM™ sequencing to screen *BRCA* genes

**DOI:** 10.7705/biomedica.5663

**Published:** 2021-12-15

**Authors:** Tiago César Gouvêa Moreira, Pricila da Silva Spínola, Micheline Campos Rezende, Carla Simone Moreira de Freitas, Fábio Borges Mury, Cibele Rodrigues Bonvicino, Luciana de Andrade Agostinho

**Affiliations:** 1 Hospital do Câncer de Muriaé, Fundação Cristiano Varella, Muriaé, Brazil Hospital do Câncer de Muriaé Fundação Cristiano Varella Muriaé Brazil; 2 Centro Universitário UNIFAMINAS, Muriaé, Brazil Centro Universitário UNIFAMINAS Muriaé Brazil; 3 Divisão de Genética, Instituto Nacional do Câncer, Rio de Janeiro, Brazil Instituto Nacional do Câncer Divisão de Genética Instituto Nacional do Câncer Rio de Janeiro Brazil; 4 Programa de Pós-Graduação em Genética, Universidade Federal do Rio de Janeiro, Rio de Janeiro, Brazil Universidade Federal do Rio de Janeiro Programa de Pós-Graduação em Genética Universidade Federal do Rio de Janeiro Rio de Janeiro Brazil; 5 Instituto de Ensino e Pesquisa Santa Casa BH, Belo Horizonte, Brazil Instituto de Ensino e Pesquisa Instituto de Ensino e Pesquisa Santa Casa BH Belo Horizonte Brazil; 6 Thermo Fisher Scientific, São Paulo, Brazil Thermo Fisher Scientific São Paulo Brazil; 7 Programa de Pós-Graduação em Neurologia, Universidade Federal do Estado do Rio de Janeiro Rio de Janeiro, Brazil Universidade Federal do Estado do Rio de Janeiro Programa de Pós-Graduação em Neurologia Universidade Federal do Estado do Rio de Janeiro Rio de Janeiro Brazil

**Keywords:** Sequence analysis, DNA, high-throughput nucleotide sequencing, genes, BRCA1, genes, BRCA2, análisis de secuencia, ADN, secuenciación de nucleótidos de alto rendimiento, genes *BRCA1*, BRCA2

## Abstract

**Introduction:**

Next Generation Sequencing (NGS) is cost-effective and a faster method to study genes, but its protocol is challenging.

**Objective:**

To analyze different adjustments to the protocol for screening the *BRCA* genes using Ion Torrent PGM sequencing and correlate the results with the number of false positive (FP) variants.

**Material and methods:**

We conducted a library preparation process and analyzed the number of FP InDels, the library concentration, the number of cycles in the target amplification step, the purity of the nucleic acid, the input, and the number of samples/Ion 314 chips in association with the results obtained by NGS.

**Results:**

We carried out 51 reactions and nine adjustments of protocols and observed eight FP InDels in homopolymer regions. No FP Single-Nucleotide Polymorphism variant was observed; 67.5% of protocol variables were jointly associated with the quality of the results obtained (p<0.05). The number of FP InDels decreased when the quality of results increased.

**Conclusion:**

The Ion AmpliSeq BRCA1/BRCA2 Community Panel had a better performance using four samples per Ion-314 chip instead of eight and the optimum number of cycles in the amplification step, even when using high-quality DNA, was 23. We observed better results with the manual equalization process and not using the Ion Library Equalizer kit. These adjustments provided a higher coverage of the variants and fewer artifacts (6.7-fold). Laboratories must perform internal validation because FP InDel variants can vary according to the quality of results while the NGS assay should be validated with Sanger.

## Resumen

**Introducción.:**

La secuenciación de nueva generación es un método rentable y rápido para el estudio de los genes, pero su protocolo entraña desafíos.

**Objetivo.:**

Investigar diferentes ajustes del protocolo de selección de los genes *BRCA* mediante secuenciación de Ion Torrent PGM™ y correlacionar los resultados con el número de variantes de falso positivo.

**Materiales y métodos.:**

El proceso de preparación de la biblioteca, el número de falsos positivos InDels, la concentración de la biblioteca, el número de ciclos en el paso de amplificación de objetivos, la pureza del ácido nucleico, la entrada y el número de muestras por chip del Ion-314 se analizaron en asociación con los resultados obtenidos por secuenciación de nueva generación secuenciación de nueva generación.

**Resultados.:**

Se hicieron 51 reacciones y nueve ajustes de los protocolos, y se observaron ocho falsos positivos InDels en las regiones de homopolímeros. No se observó ninguna variante de polimorfismo de nucleótido simple falso positivo. En 67,5 % de los casos, las variables de protocolo en su conjunto se asociaron con la calidad de los resultados obtenidos (p<0,05). El número de falsos positivos InDels disminuyó al aumentar la calidad de los resultados.

**Conclusiones.:**

El panel comunitario Ion AmpliSeq BRCA1/BRCA2 tuvo un mejor rendimiento, con cuatro muestras por chip Ion-314 en lugar de ocho, y el número de ciclos en el paso de amplificación, incluso con ADN de alta calidad, fue mejor con 23. Se observaron mejores resultados con el proceso de ecualización manual y sin el uso del kit *Ion Library Equalizer*. Estos ajustes proporcionaron una mayor cobertura de las variantes y menos artefactos. Los laboratorios deben realizar la validación interna porque las variantes de falsos positivos InDel pueden variar según la calidad de los resultados. La secuenciación de próxima generación debe validarse con Sanger.

Mutations in the *BRCA1/BRCA2* genes are associated with breast, ovarian, prostate, and pancreatic cancers ^1^, but it is not clear whether these genes increase the risk of colorectal cancer ^2^. The increasing demand for genetic testing diagnosis in a clinical setting has created the need for an alternative technology to Sanger sequencing. Next generation sequencing (NGS) is cost-effective and faster for the study of genes ^3^. However, the NGS protocol is still expensive and there are challenges throughout its steps ^4^.

NGS analysis is very useful in studies using single-nucleotide polymorphism (SNP), InDel, and genomic rearrangements ^5^. Homopolymer regions may render imprecise results, such as strand bias and low quality in determining the variants under study ^6^, while Sanger sequencing is considered the gold standard to validate mutations in these regions ^7^.

*BRCA1/BRCA2* are considered high-penetrance dominant autosomal genes for breast cancer (BC) susceptibility, and they are responsible for 25% of the risk for familial BC ^8^^-^^10^. The investigation of these genes in Brazil is important given the miscegenation of its population ^10^.

NGS is a process that requires configurations to improve analytical efficiency, as well as its sensitivity and specificity ^3^. Here we analyzed different protocol adjustments for the screening of the BRCA genes using the Ion Torrent PGM™ NGS technology and correlated the results with the number of FP variants.

## Material and methods

We conducted a transversal and observational study.

### Patients

Patients were selected from among those attending the Cancer Hospital in Muriaé city, Brazil. The inclusion criteria were the presence of breast (n=29), colorectal (n=4), rectum (n=2), prostate (n=3), and/or ovary (n=1) cancers at any age with familial or sporadic history. One negative control sample was also used. Although the association between colorectal cancer and the *BRCA1* and *BRCA2* genes is weak, we did not exclude these cases ^2^.

Eighteen years-old patients were excluded.

### Sample selection and DNA extraction

Forty peripheral blood samples were collected in two EDTA tubes. DNA extraction was performed with QIAamp® DNA Mini Kit (Qiagen) and DNA quantification was done on a Qubit 3.0 fluorometer using a dsDNA BR Assay kit while DNA qualification was done on a Nanodrop spectrophotometer (Thermo Fisher Scientific). A sample with a pathogenic variant was used as positive control and three samples were validated by the Division of Genetics at the National Cancer Institute (INCA), Brazil.

### Sample preparation and Ion Torrent PGM™ sequencing

DNA inputs were tested with 10-30 ng to construct the libraries manually using the Ion Ampliseq Library kit 2.0. We used 19, 21, and 23 cycles to amplify the targets and the Ion AmpliSeq *BRCA1* and *BRCA2* Community Panel (Thermo Fisher Scientific) with 167 primer pairs (3 primer pools). The amplicons were then partially digested, the barcodes were inserted, and the samples purified. The libraries were purified using Agencourt Ampure XP beads (Beckman Coulter), then quantified and equalized with the Ion Library Equalizer kit or manually diluted with ultrapure water. All libraries were quantified by qPCR using the Ion Library Quantitation Kit and some samples were quantified by fluorimetry using Qubit dsDNA HS Assay Kit. Multiplexed barcoded libraries were amplified by PCR emulsion using the PGM Hi-Q View Chef kit and the Ion 314 chip. The PCR emulsion was performed in an Ion Chef equipment with consecutive analysis in the PGM for the sequencing step.

### Variant selection and quality parameters

Variants were called by the Variant Caller plugin version 5.6 and compared to the genome version GRCh37/hg19 in Torrent Suite and Ion Reporter algorithms.

We tested different adjustments of protocols to screen the *BRCA1/BRCA2* genes by NGS trying different factors for the input, the library preparation process, the number of samples/Ion 314 chip, and the number of cycles in the target amplification step. The parameters evaluated at the final runs were: On target (%), mean depth (%), and uniformity and mapped reads (%) to analyze the results of the protocols performed.

We analyzed all targeted coding exons and exon-intron boundaries, as well as 20 base pairs (bp) of *BRCA1*/*BRCA2* genes. The NGS assay used is not recommended to detect large deletions and duplications variants and MLPA assay is suggested to investigate CNV variants. Here, we confirmed all pathogenic variants, some benign ones, and those of uncertain significance (VUS) by Sanger.

### Variant data analysis

Torrent Suite software version 5.2 was used to analyze the amplicons and generate quality run metrics. We used Generic-PGM-Germline Low stringency as a parameter for variant calling (cutoff with 15 reads for InDels and six for SNPs). We only selected variants with a minimum average base quality in the 30 Phred Score. They should have ≥25 when multiplying the allele coverage by the zygosity, according to the recommendation by Lih, *et al.* and the NCI- MATCH NGS assay to ensure the confidence in variant calls ^9^.

The homozygous alleles had to have a minimum value of 25 reads when the allelic frequency was 90-100%, and the heterozygous alleles, 50 reads when the frequency was 40-60% to be selected as a valid variant. All the variants with coverage below six reads were also analyzed by Sanger.

### Statistical analysis

We performed a descriptive statistical analysis using the SPSS software version 17. To compare the quality factor among the different protocols tested, we performed a non-parametric test for independent samples and multiple regression analysis to associate the quality value of each protocol tested with the library preparation process, the number of FP InDels, the library concentration in picomolar (pM), the number of cycles in the target amplification step, the 260/230 and 260/280 DNA ratios, the DNA input, and the number of samples/Ion 314 chips. We used Spearman’s test to correlate the number of InDel variants, the number of extension cycles, and the number of samples/Ion 314 chips with the quality of results. Using the independent- samples median test we compared the results obtained with the protocols. The results were considered statistically significant when p<0.05.

### Ethical approval

All the procedures involving human participants followed the Brazilian ethical standards established in Resolution 466/2012 and those by the CEP-Faminas research committee (CAAE number 62262416.3.00005105), as well as the 1964 Helsinki Declaration and later amendments or comparable ethical standards.

## Results

### Library and template preparation for NGS sequencing

Nine different protocol adjustments were done in *BRCA1/BRCA2* genes. In the first one (n=7), the sequencing chemistry used in PGM was the PGM Hi-Q Chef kit (Thermo Fisher Scientific). The target amplification step was conducted with a 19-cycle extension considering the high quality of DNA samples. In 4/7 samples we observed two amplicons with <16 reads coverage. The low coverage amplicons were AMPL225505032 and AMPL223390724 in exons 23 and 20, respectively.

In the second protocol, the extension cycle in the target amplification step (with +2) and the library concentration in pM were increased in an attempt to improve the quality of the results and the mean read length. The chemistry used was the Ion PGM Hi-Q View Chef kit. Only one of the samples analyzed in this protocol (n=29) had all amplicons covered properly; 26 samples had low coverage (<16 reads) in amplicon AMPL225505032 and 27 in AMPL223390724. Three samples had low coverage also in amplicon AMPL224626553 and one of them in AMPL225316548.

The third protocol had a cycle extension with 23 units used to amplify low- quality DNA. In 2/3 of samples analyzed, we observed a higher mean read length. All samples analyzed (n=3) had amplicons with low coverage. In 2/3 samples, the coverage stayed low in both amplicons: AMPL225505032 and AMPL223390724.

From the fourth protocol onwards, we loaded four samples in the Ion 314 chip instead of eight. All samples (n=3) had low coverage in both AMPL225505032 and AMPL223390724 amplicons. One of them, a positive control sample with a pathogenic SNP mutation, was reanalyzed in an external laboratory as reported in our methodology. In the fifth protocol, three of the samples used in protocol 2 were reanalyzed with four samples per chip and all of them had low coverage both in AMPL225505032 and AMPL223390724 amplicons.

The library preparation of protocols 1 to 5 was performed using the Ion Ampliseq Library kit 2.0 and the Ion Library Equalizer kit. It is important to mention that the number of samples analyzed per chip was different from the number of samples analyzed per protocol, the first one being only a run configuration.

In the sixth protocol, we only tested a sample already analyzed in protocol 2. The library preparation was performed following all steps in the Ion Ampliseq Library kit 2.0 including the library enrichment step with Platinum PCR SuperMix Hifi and Equalizer Primers, which differed from previous protocols. The final library concentration was approximately 77 pM and the library equalization process was manually performed with ultrapure water dilutions.

In the seventh protocol, we used one sample and the library preparation was performed using Ion Ampliseq Library kit 2.0. The enrichment step with Platinum PCR SuperMix Hifi and the Equalizer Primers was not performed. The sample was diluted to approximately 73 pM and had low coverage in both AMPL225505032 and AMPL223390724 amplicons. Mapped reads and mean depth decreased despite maintaining the same number of variants observed.

In the eighth protocol, we used two samples already investigated in protocol 2 with a larger input (20 ng/reaction); the library preparation was done according to protocol 6. Only one sample had low coverage in the amplicon AMPL223390724 and the quality factor increased.

In the ninth protocol, we analyzed the same samples used in protocol 8, but with higher input and library concentration at 30 pM. Both samples had low coverage in the AMPL223390724 amplicon and the mean read length increased. Protocols 8 and 9 are routinely applied in our molecular biology laboratory given their higher quality, absence of false positive variants, and higher mean read length of amplicons. All these adjusted protocols are shown in table 1.

**Table 1:** Detailed protocols performed and their library final concentration

Protocolo	260/230 ratio	260/280 ratio	Amplification targets (extension cycles)	Input (ng/ reaction)	Library concentration (pM) Median (min- max)	Number of samples analyzed per chip
1	1.8 (1.8-1.8)	2 (1.4-2.3)	19	20	52 (20-58)	8
2	1.8 (1.8-1.9)	2.1 (1.4- 2.3)	21	10	75 (36-111)	8
3	1.8 (1.8-1.8)	2.1 (1.9-2.2)	23	10	40 (35-44)	8
4^a^	1.8 (1.7-1.8)	2.1 (0.4-2.1)	23	10	25 (21-44)	4
5	1.8 (1.8-1.9)	2.2 (1.9-2.3)	21	10	69 (54-91)	4
6 ^a b^	1.8	2.2	23	10	76.8	4
7 ^a b^	1.8	2.1	23	10	73	4
8	1.8 (1.8- 1.9)	2 (1.9-2.2)	23	20	24 (18-30)	4
9	1.8 (1.8- 1.9)	2 (1.9-2.2)	23	30	28 (24-32)	4

^a^ One sample tested

^b^ Different manual equalization process considering the library enrichment step and the use of the Ion Library Equalizer kit

Although the same variants were observed in protocols 2 and 6 using the same sample, all quality parameters improved, except the uniformity, with approximately 95% in both runs. This sample had low coverage in both AMPL225505032 and AMPL223390724 amplicons and the mean read length increased.

The Ion NGS workflow failed to obtain an average coverage depth >20X in a small number of amplicons, but in several samples (mainly AMPL22550532 in exon 23 and AMPL223392219 in exon 20 of *BRCA2*), the IGV visual inspection and Sanger sequencing of those regions confirmed the base calls.

**Figure 1:**
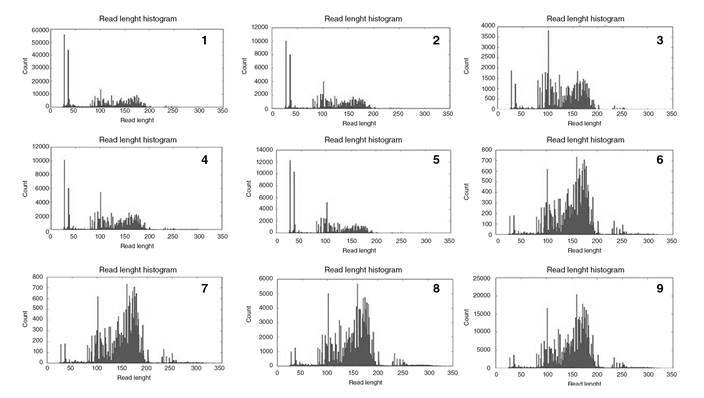
Amplicons observed in one sample as representative of each protocol

In all protocols, amplicons were lower than 50 bp (figure 1). Those smaller than 50 bp decreased when we increased the input and the number of extension cycles and when we applied four samples/chip instead of eight. We observed a 6.7-fold increase in the quality factor in protocol 9 (p<0.05) when compared to protocol 1 while with protocol 8, it was 11.3 times higher compared to protocol 1 (p=0.03).

### Protocol quality parameters

**Figure 2:**
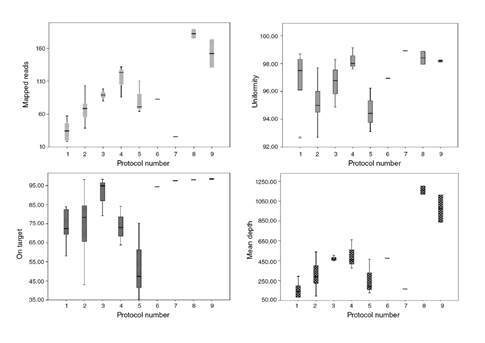
Quality parameters of samples investigated in each protocol (median value)

The quality of the results of the protocols tested was evaluated according to four variables: On target (%), mean depth (%), uniformity, and mapped reads (%) (figure 2). The 20x base coverage was constant in all protocols, from 96,6 to 99,4% (median: 99,2%), and the 100x base coverage varied from 70,3 to 99,3% (median 96,2%).

To make the statistical analysis easier, the on-target and uniformity variables were divided by 100, and to obtain the quality factor the four parameters were multiplied and divided by 1000 to generate a single quality value per protocol (table 2). All protocols had the AQ17 parameter at 100% while the AQ20 ranged from 95.5 to 97.9%. According to the base coverage, at least 96% of the bases were observed with a 20x coverage.

**Table 2:** Number and type of variants observed in each protocol (median value)

Number of protocol	n	SNP (absolute frequency)	InDel (absolute frequency)	InDel FP variants in homopolymer regions	Quality factor
1	7	11 (4-13)	2	2	12.5 (2.9-37.6)
2	29	11 (4-17)	5	5	13.5 (1.5-48)
3	3	14 (7-14)	1	1	35.5 (32.7-41.8)
4	3	8 (6-17)	0	0	35.2 (23.4-71.6)
5	3	16 (14-17)	0	0	6 (2.8-36.1)
6	1	13	0	0	35.9
7	1	12	0	0	4.1
8	2	15 (13-17)	0	0	204.4 (188.2-220.7)
9	2	15 (13-17)	0	0	146.1 (105.6-186.6)

### Variants

Among the 40 samples under study (39 affected patients and 1 negative control) distributed in 51 reactions and nine protocols, the last two had better quality and absence of FP InDel variants (table 3). The number of samples tested per protocol adjusted was not standard or larger because they were performed to validate the *BRCA1* and *BRCA2* test using NGS in our laboratory. The use of these approaches for genetic testing is complex, time-consuming, expensive, and it requires extensive technical labor. We observed 75 different variants (results not shown) in 40 samples for a total of 567 including the reproducibility test. The results obtained by Sanger detected eight FP InDels including five different variants, all of them in homopolymer regions. We should mention that not all the variants observed are described here, as we focused on the NGS assay technical field. We reported variants suspected as false positives and associated with possible artifacts given the need to improve our results and to decrease the number of FP InDels in NGS assays.

**Table 3:** False positive InDel variants included or excluded applying our validation parameters and confirmed by Sanger

Quality of InDel FP variants included by the NGS validation parameters and excluded by Sanger							
Protocol	Variant	Samples	Coding (c.)	Protein (p.)	Mean coverage	Phred score	P value
1	32906547	4	c.937_938insT	p.Ser313fs	72	356.7	0.00001
	32906565	6	c.956_957insA	p.Asn319fs	99	718.5	0.00001
2	32906535	10 e 12	c.925_926insT	p.Ser309fs	54	406.8	0.00001
	32906547	10	c.937_938insT	p.Ser313fs	50	408.8	0.00001
	32906565	1, 8, 11, 13, 17, 23, 27, 28, 29 e 30	c.956_957insA	p.Asn319fs	64	429.5	0.00001
	32906576	10 e 28	c.966_967insA	p.Val323fs	83	583.2	0.00001
	32906602	10	c.994_995insA	p.Ile332fs	51	265.7	0.00001
3	32906565	9	c.956_957insA	p.Asn319fs	124	779	0.00001
Quality of InDel FP variants excluded by the NGS validation parameters and excluded by Sanger							
1	32893197	4	c.68-17AT>A	p.?	41	63.7	0.00001
	32907302	3, 4, 6 e 7	c.1689delG	p.Trp563fs	41	365.7	0.00001
2	32907302	1, 8, 10, 11, 12, 13, 17, 18, 19, 20, 21, 22, 23, 24, 26, 28, 29, 30 e 33	c.1689delG	p.Trp563fs	57	541.5	0.00001
3	32907324	31	c.1711delT	p.Ser571fs	40	348.9	0.00001
	32907302	9	c.1689delG	p.Trp563fs	113	1079.1	0.00001
4	32907302	sg49 e 9	c.1689delG	p.Trp563fs	102	925.3	0.00001
5	32907302	21	c.1689delG	p.Trp563fs	54	488.8	0.00001
6	32907302	25	c.1689delG	p.Trp563fs	78	721.1	0.00001
8	32907302	25	c.1689delG	p.Trp563fs	314	2996.1	0.00001

Three samples were validated at the INCA laboratory (Genetics Division) by fully reanalyzing them and no contradiction between the results was seen. All variants determined as false positives were selected by the inclusion criteria and were confirmed by Sanger.

In the first protocol, we observed two InDel variants in homopolymer regions and all of them were excluded by Sanger sequencing as FP. The chemistry used in PGM was the PGM Hi-Q Ion Chef kit. In the second protocol, we observed five InDel variants, all in homopolymer regions and all considered FP. In the third protocol, we observed one FP InDel in the homopolymer region.

**Figure 3:**
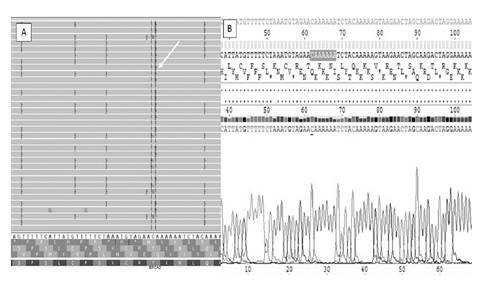
Analysis by Integrative Genomics Viewer (IGV) and by Sanger of the FP variant ch13:32906565 (GRCh37.p13). The insertion of A was observed by IGV (A) and no mutation was detected by Sanger (B).

Until protocol 3, 12 samples tested showed the rs80359770 (c.956_957insA) variant at chr13:32906565 (GRCh37.p13). The same variant was investigated by Sanger and was excluded as FP InDel despite the mean value of the Phred QUAL Score with 642.349 and 96 reads reporting an insertion of A (Ref:C, Observed Allele:CA/CA) (figure 3).

From protocol four onwards, no FP InDel was observed and the quality parameters improved. This means that when we inserted four samples/Ion 314 chip, *BRCA2* variants such as chr13:32906547 (c.937_938insT) and chr13:32906565 (c.956_957insA) were not observed again. Sample 9 was analyzed in two protocols, 3 and 4, and in this last one, the FP InDel variant disappeared.

Among the variants selected as true using the validation parameters, 29 SNPs showed coverage between 76 and 407 reads. These variants were also reanalyzed and confirmed by Sanger and none of them were in a homopolymer region.

All 8 InDels observed in protocols 1-3, with coverage of 51 to 124 reads, were in homopolymer regions. All variants were excluded as FP by Sanger and IGV analysis. In none of the 44 genetic regions (SNP) validated by Sanger, we found false-negative variants compared with NGS results.

Variants observed as no call (considering the quality score, coverage <3, and realignment errors) were validated by Sanger to confirm our laboratory’s validation parameters. No call variants were observed in chr13:32907304, 32945109, 32945115, and 32945129, and in chr17:41222985 and 41223001. These excluded variants, with strand bias and low coverage, appeared with high frequency in our sample and were not observed when analyzed by Sanger.

The parameters applied to include or exclude a variant can increase sensitivity and decrease the number of FP variants called, mainly in homopolymer regions. To solve the low coverage of some amplicons (<16) using the *BRCA1/BRCA2* panel, all samples were fully sequenced in exons 20 and 23 in the *BRCA2* gene.

Regarding the FP InDel variants included by the validation parameters and excluded by Sanger, we increased the cutoff for them. We analyzed by Sanger more than one sample bearing these InDel FP variants to find out a true threshold value considering low and high-quality runs (table 3). The InDel FP variants excluded by the validation parameters were not counted as FP variants (according to the variant data analysis described in our Materials and methods). The zygosity should also be considered to exclude a variant as FP InDel when compared with world scientific data available.

### Correlation between variants and quality parameters

The library preparation process, the number of FP InDels, the library concentration in pM, the number of cycles in the targets amplification step, the 260/230 and 260/280 DNA ratios, the DNA Input, and the number of samples/ Ion 314 chip were analyzed in association with the quality of the results. We observed that these variants jointly correlated (67.5%) to the quality of the results obtained (*R* ^*2*^ =0.67 and p<0.05).

There was a statistically significant difference between the quality of the results obtained in all the protocols tested (p=0.03). Protocols 8 and 9 showed high-quality parameters for the variables of mapped reads, uniformity, on target, and mean depth (two samples tested in each one). Protocol 9 (input with 30 ng) had more variation in mapped reads and mean depth than protocol 8 (input with 20 ng). Protocols 6 and 7 were performed with one sample each to test different equalization processes. In protocol 6 (sample 25), after the library purification step, the sample was amplified before quantification to enrich amplifiable material and to obtain a sufficient sample for accurate quantification. In protocol 7 (sample 16), after the library purification step, we diluted the sample in 50 µL of TE and did not perform the enrichment. As the enrichment step showed better quality, for the next samples we performed it. In the User guide (Thermo Fisher Scientific), this step is described in Option 3 as “Quantify the amplified library with the Qubit Fluorometer instrument”.

There was no correlation between the number of FP InDel and the quality of the results named as a quality factor (*p*=0.14), although the number of FP InDel variants decreased when the quality factor increased. No FP SNP variant was observed.

There was a weak inverse correlation of 33% between the quality of the results (quality factor) and the number of samples/Ion 314 chip (p=0.01) and a positive correlation between the quality factor and the number of extension cycles (*r*=0.50, *p*<0.05), despite DNA high quality. The number of samples per chip weakly correlated with the number of FP InDel (*r*=0.37, p=0.007).

## Discussion

The panel used in this study proved to be efficient in covering all exons and a part of the introns, but there is great variability in amplification efficiency of the 167 targets. Thus, a high value of mean coverage is essential to ensure that even regions of lower efficiency in the PCR are represented in a minimum cutoff in the sequencing data. This presentation was especially evident in our clinical cohort and confirmation by Sanger as needed for the regions with poor coverage (<20X).

The number of cycles in the amplification step for high-quality samples was 19 (as in the User guide) and, even using high-quality DNA samples, the best performance was observed with 23 cycles. The online chip calculator (Ion Ampliseq designer) recommends inserting eight samples per Ion 314 chip, but four samples per chip had better quality and fewer artifacts. We observed better quality of runs when we applied a manual equalization process and did not use of the Ion Library Equalizer kit.

The American College of Medical Genetics guidelines recommend to analyze the performance of different types of variants separately. We used Sanger to validate not only the variants with low quality parameters, but also the high-quality ones, the SNPs and the InDels, separately. This step is important for the accuracy assay and to distinguish FP from genuine variants ^11^.

InDel variants are a challenge for NGS, mainly those located in homopolymer regions ^11^^,^^12^ as the ones we observed in our study. We confirmed FP InDel variants with high quality in NGS by Sanger.

According to Park, *et al.*^13^, the quality of targeted NGS of a disease- specific subset of genes is equal to the quality of Sanger sequencing, but similarly to Bragg, *et al.* and Kang, *et al.*^11^^,^^12^, we observed some FP variants, mainly InDel, that needed validation by Sanger. In contrast to other studies, we reported all the steps of the validation process from the beginning. Different number of FP variants can be reported due to the settings used in each laboratory depending on the number of samples per chip, the use of the equalization kit, the number of amplification cycles, and the quality of samples, factors directly associated with the final quality of NGS results. A detailed validation protocol such as ours provides important information for other laboratories that are starting to use NGS sequencing so as to decrease technical errors, perform NGS validation in a shorter time interval with less cost, and avoid FP variants as candidates to be inserted in the patient report. In our study, *BRCA2* variants ch13:32906547 (c.937_938insT) and chr13:32906565 (c.956_957insA) were confirmed as FP. Mehta, *et al.* reported these variants as true germline mutations in Indian patients with breast cancer, but the coverage data and Sanger validation were not reported ^14^.

Laboratories must perform internal validation because FP variants can vary according to the run quality parameters and the presence of homopolymer regions. The selection criteria of variants must be chosen in accordance with the internal validation process of each laboratory ^15^. Lih, *et al.* strongly recommend the reprocessing from template preparation of samples with low sequencing quality. A good quality of nucleic acid samples investigated by NGS is required to obtain true results, and DNA quantification is essential ^9^.

For the Ion Ampliseq DNA library preparation in the target amplification step, the *manufacturer’s* instructions recommend an amplification cycle of 19 for high DNA quality and 22 for low quality samples. Here we used high quality samples, however, we observed greater values in quality parameters when we used 23 cycles.

The *manufacturer’s* instructions also suggest the use of Ion 314 chip with the *BRCA1/BRCA2* panel of 16 samples to obtain coverage >95% of bases at 30x. We observed that the analysis of eight samples/Ion 314 chip was not enough to obtain high coverage in some amplicons. Zanella, *et al.* also observed the AMPL22550532 in exon 23 with an average coverage depth <20x ^6^.

Studies with NGS in germline variants generally use a 20x of minimum coverage/base sequenced on both strands and accounting for at least ≥20% of the total reads ^15^^,^^16^. To solve the low coverage of some amplicons (<16 reads), we analyzed four samples/chip and the mean read length was around 151.8±1.3 bp in protocols 8 and 9. However, when eight samples/chip were analyzed, the mean read length was 110.1±11.2. Given the low coverage in some amplicons reported in our study, all samples had exons 20 and 23 sequenced by Sanger in the *BRCA2* gene.

All pathogenic variants should be confirmed by Sanger ^17^. We validated variants with low coverage and some with coverage greater than 60 reads for confirmation. Some variants with >60 read coverage were also validated because we observed an uncommon high frequency, mainly in protocols with low quality. From protocol 4 onwards, samples were re-analyzed and the FP InDel variants were not observed again.

Vendrell, *et al.* reported that some FP variants, such as *BRCA2* c.2175dup, c.1689del and c.9739del, and *BRCA1* c.5289del, had a variant allelic frequency of approximately 50% that could be attributed to systematic artifacts ^18^. Some FP variants were also described in this study (table 3), so we increased the coverage cutoff to distinguish them from the true variants. Each variant suspected as FP was checked by Sanger both in low and high- quality runs and in more than one sample to estimate the coverage cutoff mainly in homopolymer regions. Besides, the frequency of polymorphisms observed in the world population and the zygosity (in the world database Exome Aggregation Consortium - ExAC) should be considered to indicate a FP variant. If a clinical significant variant suspected as FP is observed in homozygous state by NGS and this same variant is heterozygous in the world databases, it is important to confirm with Sanger.

Jennings, *et al.* recommend Sanger sequencing coupled with targeted mutation analysis when the allele burden is expected to be low ^19^.

To obtain greater statistical power, a larger number of samples per protocol should be analyzed. In some protocols, only one sample was tested because NGS sequencing is still an expensive technique.

Beck, *et al.* analyzed 5.660 variants representing 13 unique single nucleotide variants, and 19 of them were identified by NGS but not by Sanger sequencing. They observed a minimum of 99.96% accuracy ratio for NGS compared to Sanger sequencing ^20^.

Buzolin, *et al*., used an input of 20 ng/reaction and four samples per Ion 314 chip or eight per Ion 316 chip. Using 4 samples per 314 chips they observed an average coverage with 425×, with more than 95% of the bases with a coverage of at least 100× and 98.88% at least 20×. Our eighth protocol with 20 ng/ reaction input had 189.216 mapped reads, 98.07% average base depth coverage with 1.203.000 and 98.87% of uniformity. These authors identified 587 variants, 35 of them FP (5.9%) in 26 samples analyzed ^21^. Our study found 1.4% (8/567) false positive variants considering all protocols performed and after applying the validation parameters developed.

Even after optimizing the bioinformatics parameters used in our pipeline, which improved the quality of mapping and variant calling, we had false positive variants (1.4%). The allele frequency, the zygosity, the number of studies that found similar data, and the clinical interpretation reported in databases should also be evaluated beyond the Ion Reporter™.

NGS assay offers higher throughput and a lower cost compared with Sanger ^22^. Additionally, it shows higher power estimation than Sanger ^23^ and is now being widely adopted in clinical settings ^15^. However, it requires highly complex data analysis and there are a number of challenges surrounding the technical aspects of the method ^24^^,^^25^.

The parameters under study do not include any novelty regarding the NGS assay as seen in original articles, but as a methodological paper it can help researchers involved in the complex and manual sequencing in the laboratory bench. Our analysis of the parameters is not found in scientific articles presented in a critical way and the technicians only count with the manuals of the machines as a guide in the validation step. This study helps beginners in NGS sequencing to avoid wasting time and reduces costs due to the errors generally made when using this technology for the first time.

This analytical validation met the expected performance requirements for the used intended as recommended by the manufacturer, but some important specifications should be done. The optimal number of cycles in the amplification step, even using high-quality DNA, was 23. Ion AmpliSeq BRCA1/BRCA2 Community Panel had better performance with four samples per Ion-314 chip than with eight. We observed better results when manual equalization process was done and there was no use of the Ion Library Equalizer kit. These adjustments provided higher coverage of the variants and fewer artifacts (6.7-fold in the quality of results). Laboratories must perform internal validation because FP InDel variants can vary according to the quality of results. Finally, NGS assay must be validated with Sanger in the first stage of validation in laboratories and for new and low-quality coverage variants observed in the laboratory routine (mainly InDels).
